# Supplementary material for: Cost-effectiveness of soil-transmitted helminthiasis intervention programmes: A scoping review
Source: PLoS Negl Trop Dis. 2026 May 13;20(5):e0014317. doi: 10.1371/journal.pntd.0014317 (PMC13183283; doi:10.1371/journal.pntd.0014317)
Supplement: S1 Data — (DOCX) [file pntd.0014317.s002.docx]

**Search Queries**

| **Search queries used in PubMed database** |
| --- |
| (Economic Evaluation*[tiab] OR Cost Effectiveness Analysis[tiab] OR Cost Effectiveness[tiab] OR Cost Effectiveness Ratio*[tiab] OR CEA[tiab] OR Cost Per Disability-Adjusted Life Years[tiab] OR DALYs[tiab] OR Cost-Per-DALY[tiab] OR Incremental Cost-Effectiveness Ratios[tiab] OR ICERs[tiab] OR Incremental Cost-Effectiveness Ratio[tiab] OR Quality-Adjusted Life Years[tiab] OR QALYs[tiab] OR Incremental Cost-Effectiveness Ratios[tiab] OR ICERs[tiab] OR Cost) AND ((((((((((((((((((((((((("intestinal worms"[Title/Abstract]) OR ("worm"[Title/Abstract])) OR ("worm infection"[Title/Abstract])) OR ("worm infection model"[Title/Abstract])) OR ("soil transmitted helminth"[Title/Abstract])) OR ("soil transmitted helminth ascaris"[Title/Abstract])) OR ("soil transmitted helminthiasis"[Title/Abstract])) OR ("ascaris"[Title/Abstract])) OR ("ascaris lumbricoides"[Title/Abstract])) OR ("roundworms"[Title/Abstract])) OR ("hookworms"[Title/Abstract])) OR ("ancylostoma duodenale"[Title/Abstract])) OR ("necator americanus"[Title/Abstract])) OR ("trichuris trichiura"[Title/Abstract])) OR ("trichuriasis"[Title/Abstract])) OR ("whipworms"[Title/Abstract])) OR ("Strongyloides stercoralis"[Title/Abstract])) OR ("s stercoralis"[Title/Abstract])) OR ("strongyloidiasis"[Title/Abstract])) OR ("soil transmitted helminth infections"[Title/Abstract])) OR ("human parasitic disease"[Title/Abstract])) OR ("intestinal nematode infections"[Title/Abstract])) OR ("sth"[Title/Abstract])) OR ("a lumbricoides"[Title/Abstract])) OR ("t trichiura"[Title/Abstract])). |
