# Supplementary material for: Cost-effectiveness of soil-transmitted helminthiasis intervention programmes: A scoping review
Source: PLoS Negl Trop Dis. 2026 May 13;20(5):e0014317. doi: 10.1371/journal.pntd.0014317 (PMC13183283; doi:10.1371/journal.pntd.0014317)
Supplement: Attachment — Submitted filename: PNTD-D-25-01829.docx [file pntd.0014317.s003.docx]

PLOS Neglected Tropical Diseases

**Cost-effectiveness of soil-transmitted helminthiasis intervention programmes: A scoping review**

--Manuscript Draft--

| **Manuscript Number:** | PNTD-D-25-01829 |
| --- | --- |
| **Full Title:** | Cost-effectiveness of soil-transmitted helminthiasis intervention programmes: A scoping review |
| **Short Title:** | A scoping review of soil-transmitted helminthiasis programmes |
| **Article Type:** | Research Article |
| **Keywords:** | Soil-transmitted helminthiasis; Cost-effectiveness; Mass drug administration; Scoping review |
| **Abstract:** | Background:  Soil-transmitted helminthiases (STH) affect over 1.5 billion people worldwide, causing serious health issues, especially among women of reproductive age and school-age children. Mass drug administration (MDA) using albendazole or mebendazole is the main method of control. However, high reinfection rates and limited integration with water, sanitation, and hygiene (WASH) and routine primary health care programs make the effectiveness of long-term control and elimination challenging.  Methods:  This scoping review aimed to systematically map and summarize cost-effectiveness evidence for STH control and elimination efforts. Following Arksey and O’Malley’s framework and Preferred Reporting Items for Systematic reviews and Meta-Analysis extension for Scoping Reviews (PRISMA-ScR) guidelines, we identified 22 studies published from 1993 to 2025 through PubMed and Google Scholar.  Principal findings:  Most studies focused on MDA intervention with school or community-based delivery platforms. School-based delivery costs ranged from $0.03 to $0.76 per child, and community-wide delivery costs ranged from $0.27 to $1.74 per individual. Community- wide programs were estimated to have greater impact and cost-effectiveness in high- prevalence areas, costing $28 to $198 per disability-adjusted life year averted.  Programs that delivered multiple disease interventions (integrated programs) showed the highest economic returns, primarily due to shared delivery platforms and reduced operational costs. The main factors influencing cost-effectiveness included treatment coverage, baseline prevalence, and delivery costs. Evidence gaps for cost- effectiveness still exist for preschool-aged children and integration with WASH.  Conclusion:  This scoping review showed that context affects the cost-effectiveness of STH intervention programs. Community-wide and integrated MDA strategies offered more economic value than school-based delivery in areas with high prevalence. |
| **Additional Information:** |  |
| **Question** | **Response** |
| **Government Employee**  Are you or any of the contributing authors an employee of the United States government?  Manuscripts authored by one or more US Government employees are not copyrighted, but are licensed under a [CC0](https://creativecommons.org/about/cc0) [Public Domain Dedication](https://creativecommons.org/about/cc0), which allows | No - No authors are employees of the U.S. government. |

| unlimited distribution and reuse of the article for any lawful purpose. This is a legal requirement for US Government employees.  This will be typeset if the manuscript is accepted for publication. |  |
| --- | --- |
| **Financial Disclosure**  Enter a financial disclosure statement that describes the sources of funding for the work included in this submission. Review the [submission guidelines](http://journals.plos.org/plosntds/s/submission-guidelines#loc-financial-disclosure-statement) for detailed requirements. View published research articles from [*PLOS Neglected Tropical*](https://journals.plos.org/plosntds/) [*Diseases*](https://journals.plos.org/plosntds/) for specific examples.  This statement is required for submission and **will appear in the published article** if the submission is accepted. Please make sure it is accurate. | Yes |
| **Funded studies**  Enter a statement with the following details:   - Initials of the authors who received each award - Grant numbers awarded to each author - The full name of each funder - URL of each funder website - Did the sponsors or funders play any role in the study design, data collection and analysis, decision to publish, or preparation of the manuscript? |  |
| Did you receive funding for this work? |  |
| Please add funding details.  as follow-up to "**Financial Disclosure**  Enter a financial disclosure statement that describes the sources of funding for the work included in this submission. Review the [submission guidelines](http://journals.plos.org/plosntds/s/submission-guidelines#loc-financial-disclosure-statement) for detailed requirements. View published research articles from [*PLOS Neglected Tropical*](https://journals.plos.org/plosntds/) [*Diseases*](https://journals.plos.org/plosntds/) for specific examples. | This study was conducted as part of the STOP2030 project, funded by the Global Health EDCTP3 Joint Undertaking and its members, supported by the European Union’s Horizon Europe research and innovation programme, grant number 101103089. The funders had no role in study design, data collection and analysis, decision to publish, or preparation of the manuscript. |

| This statement is required for submission and **will appear in the published article** if the submission is accepted. Please make sure it is accurate. |  |
| --- | --- |
| **Funded studies**  Enter a statement with the following details:   - Initials of the authors who received each award - Grant numbers awarded to each author - The full name of each funder - URL of each funder website - Did the sponsors or funders play any role in the study design, data collection and analysis, decision to publish, or preparation of the manuscript? |  |
| Did you receive funding for this work?" |  |
| Please select the country of your main research funder (please select carefully as in some cases this is used in fee calculation).  as follow-up to "**Financial Disclosure**  Enter a financial disclosure statement that describes the sources of funding for the work included in this submission. Review the [submission guidelines](http://journals.plos.org/plosntds/s/submission-guidelines#loc-financial-disclosure-statement) for detailed requirements. View published research articles from [*PLOS Neglected Tropical*](https://journals.plos.org/plosntds/) [*Diseases*](https://journals.plos.org/plosntds/) for specific examples.  This statement is required for submission and **will appear in the published article** if the submission is accepted. Please make sure it is accurate. | BELGIUM - BE |
| **Funded studies**  Enter a statement with the following details:   - Initials of the authors who received each award - Grant numbers awarded to each author - The full name of each funder - URL of each funder website - Did the sponsors or funders play any role in the study design, data collection and analysis, decision to publish, or preparation of the manuscript? |  |
|  |  |

| Did you receive funding for this work?" |  |
| --- | --- |
| **Competing Interests**  On behalf of all authors, disclose any competing interests that could be perceived to bias this work.  This statement will be typeset if the manuscript is accepted for publication.  Review the instructions link below and PLOS NTDs’ [competing interests](https://journals.plos.org/plosntds/s/competing-interests#loc-what-represents-a-competing-interest) policy to determine what information must be disclosed at submission. | The authors have declared that no competing interests exist. |
| **Data Availability**  Provide a **Data Availability Statement** in the box below. This statement should detail where the data used in this submission can be accessed. This statement will be typeset if the manuscript is accepted for publication.  Before publication, authors are required to make all data underlying their findings fully available, without restriction. Review our [PLOS Data Policy](https://journals.plos.org/plosntds/s/data-availability) page for detailed information on this policy. Instructions for writing your Data Availability statement can be accessed via the Instructions link below. | All relevant data are included within the paper. |

Manuscript	[Click here to access/download;Manuscript;Scoping review v6.0 -](https://www2.cloud.editorialmanager.com/pntd/download.aspx?id=1502902&guid=f149f405-a780-4447-a97d-6f20f61fbbd6&scheme=1)[<https://www2.cloud.editorialmanager.com/pntd/download.aspx?id=1502902&guid=f149f405-a780-4447-a97d-6f20f61fbbd6&scheme=1> 01112025.docx](https://www2.cloud.editorialmanager.com/pntd/download.aspx?id=1502902&guid=f149f405-a780-4447-a97d-6f20f61fbbd6&scheme=1)

# 1 Cost-effectiveness description of soil-transmitted helminthiasis intervention programmes worldwide: A scopingreview

2 Michael Odhiambo^1^, Israel Wuresah^2^, Geofrey Chepchieng^1^, Nikita Kubal^2^, Alejandro
3 Krolewiecki^3^, Charles Mwandawiro^1^, Alan Brooks^2^, Collins Okoyo^1,4*^

5

# 6 Affiliations

7 ^1^Eastern and Southern Africa Centre of International Parasite Control (ESACIPAC), Kenya
8 Medical Research Institute (KEMRI), Nairobi, Kenya
9 ^2^Bridges to Development, Geneva, Switzerland
10 ^3^Universidad Nacional de Salta, Instituto de Investigaciones de Enfermedades Tropicales/
11 CONICET, Orán, Argentina
12 ^4^Department of Epidemiology, Statistics and Informatics (DESI), Kenya Medical Research
13 Institute (KEMRI), Nairobi, Kenya

14

15	***Correspondence:** [comondi@kemri.go.ke](mailto:comondi@kemri.go.ke)

16

# 17 Abstract

18 **Background:**
19 Soil-transmitted helminthiases (STH) affect over 1.5 billion people worldwide, causing serious
20 health issues, especially among women of reproductive age and school-age children. Mass drug
21 administration (MDA) using albendazole or mebendazole is the main method of control. However,
22 high reinfection rates and limited integration with water, sanitation, and hygiene (WASH) and
23 routine primary health care programs make the effectiveness of long-term control and elimination
24 challenging.

# 25 Methods:

26 This scoping review aimed to systematically map and summarize cost-effectiveness evidence for
27 STH control and elimination efforts. Following Arksey and O’Malley’s framework and Preferred
28 Reporting Items for Systematic reviews and Meta-Analysis extension for Scoping Reviews
29 (PRISMA-ScR) guidelines, we identified 22 studies published from 1993 to 2025 through PubMed
30 and Google Scholar.

# 31 Principal findings:

32 Most studies focused on MDA intervention with school or community-based delivery platforms.
33 School-based delivery costs ranged from $0.03 to $0.76 per child, and community-wide delivery
34 costs ranged from $0.27 to $1.74 per individual. Community-wide programs were estimated to
35 have greater impact and cost-effectiveness in high-prevalence areas, costing $28 to $198 per
36 disability-adjusted life year averted. Programs that delivered multiple disease interventions
37 (integrated programs) showed the highest economic returns, primarily due to shared delivery
38 platforms and reduced operational costs. The main factors influencing cost-effectiveness included
39 treatment coverage, baseline prevalence, and delivery costs. Evidence gaps for cost-
40 effectiveness still exists for preschool-aged children and integration with WASH.

41

# 42 Conclusion:

43 This scoping review showed that context affects the cost-effectiveness of STH intervention
44 programs. Community-wide and integrated MDA strategies offered more economic value than
45 school-based delivery in areas with high prevalence.
46 **Keywords:** *Soil-transmitted helminthiasis; Cost-effectiveness; Mass drug administration;*
47 *Scoping review*

48

# 49 Author summary

50 STHs infect more than 1.5 billion people worldwide. These parasites cause anemia, poor nutrition,
51 and impaired growth and learning, particularly among children and women of reproductive age.
52 The main strategy to control STHs has long been MDA, where medicines such as albendazole
53 and mebendazole are given regularly, usually in schools and communities. While this has had a
54 significant impact on reducing the infections, reinfections are still high, especially in areas with
55 poor WASH practices. In this study, 22 published studies on the cost-effectiveness of STH control
56 programs were reviewed. School-based deworming programs cost less than community-wide
57 approaches, but community-wide programs were more cost-effective and achieved a greater
58 reduction in infections in areas with high prevalence. The study also identified important evidence
59 gaps. Few studies included preschool-aged children or women of reproductive age, and limited
60 studies assessed the impact of combining MDA with WASH interventions. Overall, the cost-
61 effectiveness of STH control varies by context. Community-wide MDA is more effective in high-
62 prevalence areas, while school-based delivery may be sufficient in lower-prevalence settings
63 depending on the dominant STH species and their transmission dynamics. The critical evidence
64 gap among Pre-SAC and WRA underscores the need to explore the integration of MDA into
65 routine primary care.

66

# 67 1.0 Introduction

68 Soil-transmitted helminthiasis (STH) remains a significant health concern, particularly in low and
69 middle-income countries (LMICs) [1]. Common STH include roundworm (*Ascaris lumbricoides*),
70 whipworm (*Trichuris trichiura*), and hookworms(*Necator americanus* and *Ancylostoma*
71 *duodenale*). They are most prevalent in sub-Saharan Africa, South Asia, and parts of Latin
72 America [1,2]. These parasites mainly infect humans through penetration of the skin by infected
73 larvae or ingestion of eggs present in contaminated soil, water, or food [3].
74 Globally, over 1.5 billion people are infected with these parasites, with women of reproductive age
75 and school-age children being particularly vulnerable to the morbidity associated with STHs, such
76 as anemia, malnutrition, impaired growth, and learning [2]. While these infections rarely cause
77 death, prolonged infection can lead to serious issues like iron deficiency (anemia), malnutrition,
78 stunted growth, and even impaired cognitive development in children, which can result in lasting
79 educational and economic disadvantages [4,5].
80 For over a decade, mass drug administration (MDA), using a single dose of albendazole or
81 mebendazole, has been the primary strategy for control of STH. These programs, often carried
82 out in schools, have significantly reduced infection rates [6]. However, reinfection remains a
83 common problem, especially in places without access to clean water, improved sanitation, and
84 hygiene education [7].
85 This ongoing challenge has sparked global interest in more comprehensive approaches. New
86 strategies for STH control and elimination aim to combine MDA with water, sanitation, and
87 hygiene (WASH) initiatives, along with health education and social behavior change. Furthermore,
88 in areas where various parasites are prevalent, the use of combined therapies, such as
89 administering ivermectin (IVM) and albendazole (ALB) together, shows promise for more
90 comprehensive and successful parasite control [8].
91 The coformulation of ALB and IVM into a single, fixed-dose combination (FDC) treatment provides
92 further evidence on the effectiveness of combined therapies. However, we still need to learn more
93 about their economic benefits through further research.
94 Although the need to improve the integration and sustainability of STH control and elimination
95 efforts is well recognized, there is notably less evidence from economic evaluations of these
96 interventions. Many studies have looked at the epidemiological impact of MDA, WASH, and
97 education strategies, but few have applied cost-effectiveness models to compare these impacts
98 with costs [9–11].
99 The few existing cost-effectiveness studies further differ in their methods, the range of
100 interventions and delivery platforms, outcome measures, and settings of implementation, which
101 makes it hard to compare them. A systematic mapping of the existing evidence is required to
102 identify what has been modeled, how it has been modeled, and the gaps remaining.
103 This scoping review aimed to gather information on the various STH interventions, analytical
104 methods, and assumptions used in STH cost-effectiveness modeling. This will help inform future
105 STH modeling studies and strengthen the economic understanding of STH elimination programs.
106 We specifically aimed to (a) identify and describe the modeling studies that evaluate the cost-
107 effectiveness of STH control and elimination programs, (b) detail the economic evaluation
108 methods used across these studies, (c) summarize the types of interventions assessed and the
109 approach of delivery, including MDA, primary health care (PHC), WASH programs, health
110 education, and integrated programs, and (d) compare the reported economic outcomes and
111 identify the specific factors that influence cost-effectiveness.

112

# 113 2.0 Materials and methods

114 A review protocol for this study was published in the Open Science Framework on September 8,
115 2025, with updates on September 28, 2025, and can be accessed at <https://osf.io/cz5xj>. The
116 writing of the manuscript of this scoping review followed the Preferred Reporting Items for
117 Systematic reviews and Meta-Analysis extension for Scoping Reviews (PRISMA-ScR) guidelines.
118 The PRISMA-ScR checklist can be found in the supplementary file (PRISMA-ScR Checklist). An
119 initial search strategy and selection criteria were developed to guide the development of this
120 scoping review. We used the five-step scoping review process that Arksey & O’Malley [12]
121 proposed, which included research question identification, study identification, relevant study
122 selection, data extraction, and result presentation.

# 123 2.1 Review questions

124 Given the need to strengthen the economic evidence for STH control and elimination programs,
125 this scoping review mapped and described modeling studies that looked at the cost-effectiveness
126 of STH interventions. The aim was to indicate the types of interventions studied, the modeling
127 methods used, and the factors that affect the reported economic results. This was intended to
128 inform future modeling efforts and support the economic case for including FDC treatment in STH
129 control and elimination strategies.

# 130 2.2 Study identification

131 Study identification search was based on two databases, PubMed and Google Scholar, for
132 published records or studies on cost and cost-effectiveness using a predefined search strategy.
133 We included the Boolean logic operators “AND” (to return results when both keywords are met)
134 and “OR” (to return results when at least one of the keywords is met), together with keywords
135 such as cost, cost-effectiveness, and names of diseases of interest (soil-transmitted
136 helminthiasis, soil-transmitted helminths, STH, *Ascaris*, *Ascaris lumbricoides*, *A. lumbricoides*,
137 hookworm(s), worm(s), roundworms, *Ancylostoma duodenale*, *Necator americanus, Trichuris*
138 *trichura*, *T. trichura*, whipworm(s), *Strongyloides stercoralis, S.* s*tercoralis*, human parasitic
139 disease, and intestinal nematode infections) in our search strategy. An advanced search was
140 conducted in PubMed using the search queries attached as Appendix I. The queries were then
141 modified to suit the Google Scholar search engine.

# 142 2.3 Eligibility, screening, and selection criteria

143 Studies that performed economic analyses, specifically cost-effectiveness models or combined
144 cost and cost-effectiveness analyses, on STH were included. This includes intestinal worms,
145 *Strongyloidiasis*, and other STH infections listed in the search query. Eligible studies were
146 published in English, did not include protocols or reviews, and examined preventive
147 chemotherapy interventions such as albendazole, mebendazole, levamisole, or ivermectin, or
148 other auxiliary interventions like WASH, PHC, health education, social behaviour change (SBC),
149 or integrated programs. We placed no restrictions on publication year or geographical location.
150 All records were imported into Mendeley Reference Manager to remove duplicates. Screening
151 took place in two stages. First, we reviewed titles and abstracts to identify studies focused on
152 cost, cost-effectiveness, economic modeling, or related economic evaluations. We excluded
153 records that did not meet the inclusion criteria. Second, four reviewers independently assessed
154 the full texts to confirm each study’s relevance to the specified diseases and interventions. We
155 resolved discrepancies through discussion and sought periodic input from senior reviewers. Final
156 inclusion followed the PRISMA-ScR guidelines [13]. Table 1 shows the rationale and criteria for
157 eligibility of including studies in this review.
158 ***Table 1: Eligibility criteria for publications in the review and the rationales for selecting these***
159 ***criteria***

| **Category** | **Criterion (inclusion: white, exclusion:**  **orange** | **Rationale** |
| --- | --- | --- |
| **Population** | Studies conducted on human populations (all ages, including children, adults, women of reproductive age, and communities). | The review is concerned with the cost-effectiveness of interventions targeting STH  infections among humans. |
|  | Studies conducted on non-human  populations (e.g., animal-only studies, lab- only models). | This review is not concerned  with studies targeting non- human populations. |
| **Concept** | Studies that perform economic analyses, specifically cost-effectiveness, or that combine cost and cost-effectiveness analyses of STH interventions. | This review targeted studies that performed economic evaluations of STH  interventions to guide policy and program design. |
|  | Studies not addressing STH; studies without cost, cost-effectiveness, or economic evaluation metrics; studies only epidemiological or clinical without economic modeling. | This review aims to map economic evidence for soil- transmitted helminthiasis interventions. Studies not addressing STH are outside  the disease scope. |
| **Context** | Global scope; no restriction on country or region. | STH is a global health problem,  so all geographical regions were considered relevant. |
| **Interventions** | Preventive chemotherapy (albendazole, mebendazole, levamisole, ivermectin); auxiliary interventions (WASH, PHC, health education, social behavior change,  integrated programs). | Review targeted both pharmaceutical and integrated interventions relevant to STH. |
|  | Studies without preventive chemotherapy or auxiliary interventions related to STH control/elimination. | This review focused specifically on interventions with direct or indirect impact on STH  control/elimination. |

| **Study Design / Evidence Sources** | Primary studies (modeling studies, field studies, economic evaluations) published in English; peer-reviewed. | Ensured inclusion of studies with extractable cost- effectiveness data, excluding secondary or inaccessible  sources. |
| --- | --- | --- |
|  | Protocols, reviews, commentaries, non-  English studies, incomplete or restricted access papers. | These do not provide  completed economic analyses or results. |

160

# 161 2.3.1 Quality assessment of the extracted records

162 Although established critical appraisal tools for economic evaluations exist, such as the
163 Drummond checklist and JBI, in keeping with the objectives of this review, we did not apply these
164 formal tools. Instead, during the full-text review and data extraction process, we took into account
165 descriptive indicators of quality, such as records with accessible cost-effectiveness metrics (e.g.,
166 Incremental Cost-Effectiveness Rations (ICERs), Disability-Adjusted Life Years (DALYs) averted,
167 unambiguous economic evaluation techniques, and relevance to the targeted helminths and the
168 interventions evaluated.

# 169 2.4 Data extraction and reporting

170 The data extraction was conducted systematically using a consistent method by four reviewers,
171 following the decision on what information to extract. Disagreements were discussed and resolved
172 among the reviewers with the help of two other subject-level experts. The following details were
173 gathered from the records: author name, publication year, title, location (country/WHO region),
174 target group/population, target helminth, methods, outcome metrics, transmission parameters,
175 cost-effectiveness parameters, intervention, comparison group, data types, and key results. This
176 information was then checked to ensure its accuracy and consistency across each record. We
177 entered the extracted information into a template we created in Microsoft Excel for descriptive
178 analysis.

# 179 2.4.1 Outcomes and prioritization

180 The primary outcomes extracted from the included studies were economic indicators, specifically
181 ICERs, DALYs averted, and cost per infection or morbidity reduction. These outcomes were
182 prioritized due to their critical relevance for guiding resource allocation and informing public health
183 policy decisions, particularly in settings with constrained resources and persistent STH
184 transmission. Additionally, this scoping review aimed to identify key parameters, assumptions,
185 and methodological approaches commonly used in existing economic evaluations. Mapping these
186 elements are essential for the future development of a comprehensive and robust cost-
187 effectiveness model. Such a model would explicitly evaluate the economic feasibility of novel
188 interventions, including simplified FDC therapies, and assess their suitability for broader target
189 populations such as preschool-aged children and adults.

# 190 2.5 Data analysis

191 The analysis followed Arksey & O’Malley [12] framework for gathering, summarizing, and
192 reporting scoping review results. We extracted relevant information from the final set of records
193 and analyzed it descriptively to look at its nature, distribution, and scope. We visualized the
194 findings in tables and graphs that show the geographic distribution of studies, types of
195 interventions, target populations, target helminths, and methods of analysis. We grouped
196 outcomes into common themes, patterns, and trends to synthesize the results. All analyses were
197 done in Microsoft Office Excel version 16.100.3.

# 198 3.0 Results

199 **3.1 Description of study selection**
200 Drawing on the Arksey and O’Malley framework, we synthesized findings from twenty-two studies,
201 mapping their study characteristics, methods, and outcomes, while also identifying critical gaps
202 in the evidence base. A PRISMA flow diagram (Figure 1) summarizes the stages of the study
203 selection process, from identification through screening and final inclusion. The included studies
204 represented a broad geographical distribution, with the majority of the studies conducted in sub-
205 Saharan Africa, followed by East and Southeast Asia, and a smaller number from Latin America.
206 Several analyses were modeling-based and not tied to specific field sites.

207

208

209 ***Figure 1: PRISMA flow diagram showing the identification, screening, and inclusion***
210 ***processes***

211

# 212 3.2 Target Populations, Geographic Scope, and Intervention Modalities

213 Table 2 outlines the details extracted from each study, which includes: the author, publication
214 year, geographic location, primary research focus, target population, type of intervention
215 assessed, and key economic outcomes reported.

216

217

218	**Table 2: Summary characteristics of studies included in the scoping review**

| **Author, year** | **Location**  **(Country/Region)** | **Focus** | **Population** | **Intervention** | **Economic**  **Outcome** |
| --- | --- | --- | --- | --- | --- |

| Zhu et al., 2025 [14] | China (Western Pacific) | System dynamics modelling for hookworm in China | Whole population | WPD^1^, KPD^2^, ED^3^, EVD^4^ +  health education | ICER^5^ per infection reduced |
| --- | --- | --- | --- | --- | --- |
| Minnery et al., 2024 [15] | Kenya (Africa) | Survey cost- effectiveness for  Kenyan deworming | Schoolchildren | Geostatistical vs cluster  survey | ICER per survey  precision |
| Coffeng et al., 2024 [16] | Not country- specific | MDA ivermectin for strongyloidiasis | Children 5-15  and community 5+ years | Annual ivermectin MDA | Cost/DALY^6^ averted |
| Coffeng et al., 2023 [17] | Ethiopia and Tanzania (Africa) | Diagnostic cost- effectiveness framework | Schoolchildren | Diagnostic tools cost- effectiveness | Cost per reliable diagnostic  result |
| Trinos et al., 2023 [18] | Vietnam and Philippines (Western Pacific) | MDA for hookworm in Vietnam and Philippines | Entire community including  preschool aged children | MDA with Albendazole | ICER per DALY and infection-  years averted |
| Cha et al., 2019 [19] | Sudan (Africa) | Nationwide STH and schistosomiasis  survey in Sudan | School children | Targeted ecological MDA | Efficiency of ecological vs district-  level MDA |
| Pullan et al., 2019 [7] | Kenya (Africa) | Community-wide vs school-based MDA in Kenya | Children 2-14 and community 2+ years | Annual vs biannual MDA | Cost/person and prevalence  reduction |
| De Neve et al., 2018 [20] | Madagascar (Africa) | Integrated PC in Madagascar | School-aged children | School-based PC^7^, integrated NTDs | ICER per DALY,  benefit-cost  ratios |
| Okello et al., 2018 [21] | Northern Lao PDR (Western Pacific) | One Health integration: *T.*  *solium* + STH + swine fever | Communities & pigs | Integrated One Health MDA & pig vaccination | Cost/DALY and zDALY^8^ averted |
| Thakur et al., 2018 [22] | Ghana (Africa) | Optimizing MDA by age/risk in Ghana | Children & adults (risk  stratified) | MDA by age/risk mix | Cost/infectio n reduction  per strategy |
| Lo et al., 2016 [23] | Côte d’Ivoire (Africa) | Global MDA guideline assessment | Children, preschool, adults | Annual/biannual PC, integrated | ICER per DALY,  prevalence  thresholds |
| Bartsch et al., 2016 [24] | Brazil (Latin America) | Hookworm vaccine  + MDA in Brazil | Children & adults | MDA vs vaccine | ICER for  vaccine & MDA |
| Turner, et al., 2016 [25] | Uganda (Africa) | Economies of scale in Uganda MDA | Schoolchildren | Scaling MDA with economies | Cost- effectivenes s under  economies of scale |

| Turner, Truscott, &  Anderson, 2016 [26] | Côte d’Ivoire (Africa) | Community-wide vs school-only  MDA in Côte d'Ivoire | Entire community | Community- wide MDA vs school | ICER per DALY  averted |
| --- | --- | --- | --- | --- | --- |
| Lo et al., 2015 [27] | Côte d’Ivoire (Africa) | Community-wide integrated PC in Côte d'Ivoire | Community- wide, all ages | Integrated community-wide PC | ICER per DALY  averted robust to  high costs |
| Evans et al., 2011 [28] | Nigeria (Africa) | Triple-drug MDA Nigeria | School-aged children | Triple-drug MDA  (ivermectin, albendazole, and  praziquantel) | Cost savings per treatment delivered |
| Leslie et al., 2011 [29] | Niger (Africa) | School vs community MDA in Niger | Children & adults | School vs community MDA | Cost/infectio n averted, children vs  adults |
| Hall et al., 2009 [30] | Africa, Asia, Latin America | MDA thresholds cost-effectiveness | Preschool & school-aged children | WHO vs 3-tier thresholds | Cost/diseas ed person treated |
| Brooker et al., 2008 [31] | Uganda (Africa) | Nationwide school- based MDA in  Uganda | Schoolchildren | Nationwide school MDA | ICER per anemia  averted |
| Guyatt, 2003  [32] | Kenya (Africa) | Program delivery cost in Kenya | Schoolchildren & adults | School vs  community delivery | Cost per  treatment, affordability |
| Holland et al., 1996 [33] | Nigeria (Africa) | Levamisole MDA in Nigeria | Community (children focus) | Mass, targeted, selective MDA | Cost per 1,000 epg  reduced |
| Guyatt et al., 1993 [34] | Model-based (not location-specific) | Dynamic CEA Ascaris treatment frequency | Community- wide | Annual, biannual MDA | Cost per case  averted by frequency |

219

220

221

222

223

224

225

226

227

228

229

230

231

^1^Whole population deworming ^2^Key population deworming ^3^Examination-and-deworming ^4^Examination and voluntary deworming ^5^Incremental cost-effectiveness ratio ^6^Disability-adjusted life years ^7^Preventive chemotherapy ^8^Zoonotic Disability-adjusted life years

# 3.2.1 Geographical Distribution

Across the 22 studies, sub-Saharan Africa accounted for the largest share of evidence (59%, n=13), with particularly high representation from Kenya (n=3) and Côte d’Ivoire (n=3), followed by Uganda (n=2), Nigeria (n=2), Madagascar (n=1), Niger (n=1), and Ghana (n=1). Southeast/East Asia contributed 18% (n=4), including China, Vietnam, the Philippines, and Lao PDR. Latin America was represented by a single study from Brazil (5%). Figure 2 presents a geographical map showing the countries represented in the included studies, while noting that three studies were excluded from the figure because they were simulation-based and not tied to a specific country.

232

233

234 ***Figure 2:*** *Geographic distribution of included studies. Countries are shaded according to the*
235 *number of studies conducted in each location. Three simulation-based studies that did not*
236 *correspond to a specific country are not shown.*

# 237 3.2.2 Target Populations

238 Most programs focused on school-aged children (41%, n=9) (Figure 3), reflecting WHO
239 guidelines. A larger proportion (59%, n=13) expanded to entire communities, including adults,
240 women of reproductive age, and in some cases, preschool-aged children, with evidence that this
241 expansion improves cost-effectiveness in moderate to high prevalence settings [7,18,27].

242

243

***Figure 3:*** *Distribution of target populations among included studies*

13

School-aged children

Entire community

9

**S c h o o l - a g e d c h i l d r e n**

**E n t i r e c o m m u n i t y**

# 244 3.2.3 Types of Intervention

245 The majority of studies (68%, n=15) examined MDA, either school-based, community-wide, or
246 integrated into neglected tropical disease (NTD) programs. School-based MDA programs were
247 delivered at low cost, ranging from $0.03 to $0.76 per child treated, and were often integrated
248 with other NTD control activities to improve efficiency (Brooker et al., 2008; De Neve et al., 2018;
249 Leslie et al., 2011). Evidence from Uganda and other settings demonstrated that economies of
250 scale (the cost advantage that arises with increased output, where the average cost per unit
251 decreases as the number of people treated increases [35]) significantly reduced per-child costs
252 as coverage expanded [26,31].
253 Community-wide MDA interventions were frequently more cost-effective per DALY averted than
254 school-based approaches, particularly in high-prevalence settings with substantial adult infection
255 burdens. Reported incremental cost-effectiveness ratios (ICERs) for these programs ranged from
256 $28.55 to $198 per DALY averted [16,18]. Expanding treatment beyond school-aged children
257 increased the overall impact on transmission and improved cost-effectiveness.
258 Integrated or multi-disease delivery models also showed strong economic performance. In
259 Nigeria, bundling ivermectin, albendazole, and praziquantel into a single delivery platform
260 reduced per-person delivery costs by 41% [28]. Favorable DALY and benefit-cost ratios were also
261 achieved in Lao PDR and Madagascar [20,21]. One study modeled the combination of a
262 hookworm vaccine with MDA in Brazil, aiming to reduce reinfection rates and extend deworming
263 benefits [24].

264

265

# 266 3.3 Economic Outcomes and Methodological Approaches

267 **3.3.1 Economic Metrics**
268 Cost per disability-adjusted life year (DALY) averted was the most common outcome, reported in
269 45% of studies, with values ranging from US$14 for a One Health program in Lao PDR to US$198
270 for school-based preventive chemotherapy targeting strongyloidiasis. Incremental cost-
271 effectiveness ratios (ICERs) were widely used; for example, community-wide versus school-only
272 MDA in Côte d’Ivoire yielded ICERs between US$127 and US$167 per DALY averted [26,27].
273 Cost per treatment delivered varied substantially by delivery mode. School-based programs
274 ranged from US$0.03 to US$0.76, while community-based programs ranged from US$0.27 to
275 US$1.74 per treatment. In community-wide contexts, cost per infection-year averted was often
276 under US$1 [18]. Benefit-cost ratios reached as high as US$31 for integrated school-based
277 programs [20].

# 278 3.3.2 Methodological diversity

279 Studies applied diverse modeling approaches. Dynamic transmission models were used in 32%
280 of the studies (n=7) [22,23,25,25,27,34,36], supporting expansion of MDA to preschool-aged
281 children and adults, even under higher assumed delivery costs [27]. Program delivery cost
282 analyses, accounted for 32% of the studies (n=7), highlighted affordability drivers and economies
283 of scale. Other frameworks included Markov models (5%, n=1), system dynamics models (5%,
284 n=1), and stochastic individual-based simulations (5%, n=1), which confirmed robustness across
285 assumptions for cure rates, delivery costs, and time horizons [16].

# 286 3.4 Cross-cutting findings

287 **3.4.1 Recurring patterns**
288 Community-wide MDA consistently outperformed school-only strategies in DALYs and infection-
289 years averted. Integrated platforms addressing multiple diseases produced the highest returns
290 per dollar spent. Programs benefited from economies of scale, with per-person costs declining as
291 coverage expanded.

# 292 3.4.2 Key drivers of cost-effectiveness

293 Sensitivity analyses were reported in 9 of 22 studies (44%), most commonly testing the impact of
294 drug delivery costs, baseline prevalence levels, and treatment coverage on cost-effectiveness
295 outcomes. For example, Lo et al. showed that community-wide treatment strategies remained
296 highly cost-effective when adult delivery costs were assumed to be ten times higher than those
297 for children [27]. Coffeng et al. [16] demonstrated that community-wide ivermectin MDA
298 consistently remained the preferred strategy under a wide range of assumptions for cure rates,
299 delivery costs, time horizons, and coverage. Similarly, Trinos et al. used probabilistic sensitivity
300 analysis to confirm the robustness of community-wide MDA in Vietnam and the Philippines. Other
301 studies, including De Neve et al. [20], Okello et al. [21], Bartsch et al. [36], and Brooker et al. [31]
302 incorporated parameter uncertainty or scenario analyses, highlighting the role of program costs,
303 drug efficacy, and prevalence thresholds as key drivers. Collectively, these analyses strengthen
304 confidence in the conclusion that community-wide and integrated approaches tend to outperform
305 school-based MDA, though the lack of sensitivity analyses in over half of the studies limits cross-
306 study comparability and generalizability.

# 307 3.4.3 Gaps in the evidence

308 Geographic gaps remain, with limited evidence from the Western Pacific, the Caribbean, and
309 most of Latin America. Few studies included preschool-aged children, and none considered
310 children under two years. Analyses of fixed-dose combination therapies are scarce, likely due to
311 their novelty. Evidence on long-term sustainability, integration with WASH, and broader One
312 Health approaches is also limited. Diagnostic and surveillance-focused evaluations rarely
313 reported disability-adjusted based outcomes, reducing comparability with treatment-focused
314 analyses. Also, only one study specifically addressed *Strongyloides stercoralis* [16].

# 315 4.0 Discussion

316 This scoping review revealed that community-wide and integrated MDA approaches tend to be
317 more cost-effective than school-based delivery in high-prevalence settings, particularly when
318 combined with other NTD programs and scaled to larger populations.
319 The ICER reported by the studies ranged from US$14 to US$198 per DALY averted. The variation
320 was largely due to differences in coverage levels, integration, and baseline prevalence. That is,
321 the cost-effectiveness of STH interventions is highly context-specific, influenced by delivery
322 strategy, population coverage, and program integration. In high-prevalence settings, community-
323 wide MDA was more impactful than school-based delivery because adult treatment interrupts
324 transmission, but reinfection limited overall effects [18,27]. However, the operational feasibility of
325 such approaches relies on the ability to reach geographically reduced infected adult populations
326 and health systems’ willingness for higher upfront costs. Integrated NTD campaigns consistently
327 improved cost-effectiveness by sharing delivery platforms and reducing per-person costs
328 [21,28].
329 Further, we found uneven amounts of data on cost-effectiveness from endemic regions. Most
330 studies focused on Sub-Saharan Africa, with limited evidence emerging from other endemic
331 regions like Latin America, the Western Pacific, or the Caribbean. These gaps are particularly
332 consequential because economic efficiency does not solely rely on biological impact. It also
333 incorporates local delivery costs, local health system capacity, baseline prevalence patterns, and
334 other factors [7]. With a limited geographical scope, policies are likely to be centered on strategies
335 tailored to scenarios with mismatched epidemiological or economic attributes.
336 Additionally, differences in methodological approaches were noticed across the studies included
337 in this review, which makes comparability difficult. Expanded coverage scenarios were often
338 associated with more favorable cost-effectiveness ratios in dynamic transmission models, while
339 static cost analyses were more centered on near-term affordability and gave a narrower view.
340 Inconsistent parameter assumptions, especially concerning cure rates, delivery costs, and time
341 frames, make cross-study synthesis challenging as well. The interpretability and relevance of
342 policies derived from Cost-Effectiveness (CE) studies could be improved with more uniform
343 modeling approaches combined with clear sensitivity analyses [16].
344 One major gap we found in our review is the lack of evidence and intervention among important
345 at-risk groups. Preschool-aged children and WRA are, in general, underrepresented in economic
346 evaluations, and cost-effectiveness analyses (CEAs) conducted to date also seem to have
347 completely ignored these critical groups. This gap is concerning in that these groups are
348 especially susceptible to morbidity of STH infections, including stunted growth and development,
349 among Pre-SAC, and anemia [4]. In the absence of economic evidence for these children,
350 policymakers are less able to budget resource allocation. This makes it more difficult to design
351 targeted interventions for some of the most at-risk groups, ultimately undermining efforts to
352 eliminate STH at the population level.
353 A key gap in the literature is the limited examination of the long-term sustainability and cost-
354 effectiveness of interventions implemented without concurrent WASH improvements. Many
355 studies evaluate the immediate outcomes of mass drug administration (MDA) but fail to assess
356 whether the benefits persist, particularly if reinfection rates return to baseline once treatment
357 stops. Sustainable reduction of STH infections requires not only consistent access to medication
358 but also significant environmental improvements that lower the risk of reinfection. The failure to
359 evaluate the combination of WASH with MDA in the long term suggests that these strategies could
360 be only temporally effective. The variability in context and delivery costs across countries was
361 also not consistently accounted for in the included studies. This limits the comparability of findings
362 across settings and highlights the need for future analyses to explicitly incorporate local economic
363 and health system factors when modeling cost-effectiveness.
364 This scoping review is particularly important for assessing the possible cost-effectiveness of a
365 new FDC of albendazole and ivermectin for STH. Integrated MDA campaigns support the claim
366 that treatments, when combined and delivered in a single delivery event, could significantly
367 reduce cost per person and increase the return on investment. This is particularly true in areas
368 where STH epidemiology calls for both drugs to be used [8]. The FDC’s broader activity could
369 achieve a greater health impact by concurrently eliminating STH and other parasites, thus
370 increasing the DALYs averted per treatment round. Nonetheless, without empirical cost-
371 effectiveness estimates for FDC, modeling will need to account for potential gains in efficiency
372 against uncertainties in manufacturing cost, procurement pricing, and adoption feasibility. The
373 literature suggests that such an intervention would likely be most cost-effective in high-
374 prevalence, co-endemic settings where community-wide coverage is feasible and integration with
375 existing NTD programs can be achieved.
376 From the reviewed studies, there was a notable absence of costs associated with monitoring and
377 evaluation (M&E) frameworks in the cost-effectiveness analyses. Robust M&E systems are
378 essential to track program coverage, impact, treatment compliance, reinfection dynamics, and the
379 sustainability of the interventions. Integrating M&E costs into cost-effectiveness models would
380 complete the intervention cycle and enable more accurate policy guidance and resource
381 allocation.
382 Nevertheless, this review has some notable limitations. First, the reliance on English-language
383 publications may have excluded relevant evidence from non-English sources, notably literature
384 from the Americas published in Spanish. Second, the heterogeneity of economic metrics,
385 modeling assumptions, and intervention designs across studies limited our ability to perform direct
386 quantitative comparisons. Finally, given the inclusion of studies spanning more than three
387 decades, differences in global and local economic conditions, inflation, drug pricing, and
388 healthcare infrastructure could influence the comparability of cost-effectiveness results. As a
389 scoping review, we did not perform temporal standardization or inflation adjustments. Therefore,
390 findings should be interpreted as relative rather than absolute economic measures.
391 In conclusion, this scoping review demonstrates that context has an impact on the cost-
392 effectiveness of STH interventions, with community-wide and integrated MDA strategies typically
393 providing higher economic value than school-based delivery in high-prevalence settings.
394 Improved efficiency was consistently driven by utilizing economies of scale and integrating with
395 other NTD control activities. However, there is uncertainty regarding the generalizability of the
396 findings of this study to other endemic regions due to the fact that the evidence base of the
397 included studies was heavily concentrated in sub-Saharan Africa. Cross-study comparability is
398 further restricted by methodological diversity and inconsistent parameter assumptions. The
399 review also identifies important evidence gaps for interventions that combine environmental and
400 pharmaceutical approaches, as well as for high-risk but underrepresented groups, such as Pre-
401 SAC and WRA.
402 The findings carry direct relevance for assessing the potential cost-effectiveness of the FDC of
403 albendazole and ivermectin. Experience from integrated MDA campaigns suggests that such a
404 formulation could improve efficiency and impact, particularly in areas with multiple STH burden
405 and high-prevalence contexts where community-wide coverage is achievable. Nonetheless,
406 empirical economic evaluations of FDC for STH control are lacking, highlighting the need for
407 robust modeling and field-based studies to confirm these projections.

# 408 Financial disclosure

409 This study was conducted as part of the STOP2030 project, funded by the Global Health EDCTP3
410 Joint Undertaking and its members, supported by the European Union’s Horizon Europe research
411 and innovation programme, grant number 101103089. The funders had no role in study design,
412 data collection and analysis, decision to publish, or preparation of the manuscript.

# 413 Competing interests

414 The authors have declared that no competing interests exist.
415 **Data availability statement**

416

417

All relevant data are included within the paper.

# 418 References

419 1. Brooker S, Clements ACA, Bundy DAP. Global Epidemiology, Ecology and Control of Soil-
420 Transmitted Helminth Infections. Adv Parasitol. 2006;62:221–61. https://doi.org/10.1016/S0065-
421 308X(05)62007-6
422 2.	WHO.	Soil-transmitted	helminth	infections	[Internet].	2023	[cited	2025	Mar	4].
423 https://[www.who.int/news-room/fact-sheets/detail/soil-transmitted-helminth-infections.](http://www.who.int/news-room/fact-sheets/detail/soil-transmitted-helminth-infections) Accessed
424 4 Mar 2025
425 3. Mbong Ngwese M, Prince Manouana G, Nguema Moure PA, Ramharter M, Esen M, Adégnika
426 AA. Diagnostic Techniques of Soil-Transmitted Helminths: Impact on Control Measures. Trop
427 Med	Infect	Dis.	Multidisciplinary	Digital	Publishing	Institute;	2020;5:93.
428 https://doi.org/10.3390/tropicalmed5020093
429 4. Freeman MC, Akogun O, Jr VB, Brooker SJ, Gyorkos TW, Imtiaz R, et al. Challenges and
430 opportunities for control and elimination of soil-transmitted helminth infection beyond 2020. PLoS
431 Negl	Trop	Dis.	Public	Library	of	Science;	2019;13:e0007201.
432 https://doi.org/10.1371/journal.pntd.0007201
433 5. Hotez PJ, Bottazzi ME, Franco-Paredes C, Ault SK, Periago MR. The neglected tropical
434 diseases of Latin America and the Caribbean: A review of disease burden and distribution and a
435 roadmap	for	control	and	elimination.	PLoS	Negl	Trop	Dis.	2008;2.
436 https://doi.org/10.1371/JOURNAL.PNTD.0000300
437 6. Collyer BS, Turner HC, Hollingsworth TD, Keeling MJ. Vaccination or mass drug administration
438 against schistosomiasis: A hypothetical cost-effectiveness modelling comparison. Parasit
439 Vectors.	BioMed	Central	Ltd.;	2019;12:1–14.	https://doi.org/10.1186/S13071-019-3749-
440 4/FIGURES/6
441 7. Pullan RL, Halliday KE, Oswald WE, Mcharo C, Beaumont E, Kepha S, et al. Effects, equity,
442 and cost of school-based and community-wide treatment strategies for soil-transmitted helminths
443 in Kenya: a cluster-randomised controlled trial. Lancet Lond Engl. England; 2019;393:2039–50.
444 https://doi.org/10.1016/S0140-6736(18)32591-1
445 8. Krolewiecki A, Kepha S, Fleitas PE, Lieshout L van, Gelaye W, Messa A, et al. Albendazole–
446 ivermectin co-formulation for the treatment of Trichuris trichiura and other soil-transmitted
447 helminths: a randomised phase 2/3 trial. Lancet Infect Dis. Elsevier; 2025;25:548–59.
448 https://doi.org/10.1016/S1473-3099(24)00669-8
449 9. Anderson RM, Truscott JE, Pullan RL, Brooker SJ, Hollingsworth TD. How effective is school-
450 based deworming for the community-wide control of soil-transmitted helminths? PLoS Negl Trop
451 Dis. United States; 2013;7:e2027. https://doi.org/10.1371/journal.pntd.0002027
452 10. Fitzpatrick C, Fleming FM, Madin-Warburton M, Schneider T, Meheus F, Asiedu K, et al.
453 Benchmarking the Cost per Person of Mass Treatment for Selected Neglected Tropical Diseases:
454 An Approach Based on Literature Review and Meta-regression	with Web-Based Software
455 Application.	PLoS	Negl	Trop	Dis.	United	States;	2016;10:e0005037.
456 https://doi.org/10.1371/journal.pntd.0005037
457 11. Turner HC, Truscott JE, Hollingsworth TD, Bettis AA, Brooker SJ, Anderson RM. Cost and
458 cost-effectiveness of soil-transmitted helminth treatment programmes: systematic review and
459 research needs. Parasit Vectors. England; 2015;8:355. https://doi.org/10.1186/s13071-015-
460 0885-3
461 12. Arksey H, O’Malley L. Scoping studies: towards a methodological framework. Int J Soc Res
462 Methodol. Routledge; 2005;8:19–32. https://doi.org/10.1080/1364557032000119616
463 13. Tricco AC, Lillie E, Zarin W, O’Brien KK, Colquhoun H, Levac D, et al. PRISMA Extension for
464 Scoping Reviews (PRISMA-ScR): Checklist and Explanation. Ann Intern Med. American College
465 of Physicians; 2018;169:467–73. https://doi.org/10.7326/M18-0850
466 14. Zhu H, Zheng J, Huang J, Zhang M, Zhou C, Zhu T, et al. Optimal control strategies supported
467 by system dynamics modelling: a study on hookworm disease in China. Infect Dis Poverty.
468 BioMed Central Ltd; 2025;14. https://doi.org/10.1186/s40249-025-01293-w
469 15. Minnery M, Okoyo C, Morgan G, Wang A, Johnson O, Fronterre C, et al. Cost-effectiveness
470 of comparative survey designs for helminth control programs: Post-hoc cost analysis and
471 modelling of the Kenyan national school-based deworming program. PLoS Negl Trop Dis. United
472 States; 2024;18:e0011583. https://doi.org/10.1371/journal.pntd.0011583
473 16. Coffeng LE, Lo NC, Vlas SJ de. Cost-effectiveness of mass drug administration with
474 ivermectin	against	strongyloidiasis:	a	modelling	study	[Internet].	medRxiv;	2024.
475 https://doi.org/10.1101/2024.04.04.24305312
476 17. Coffeng LE, Vlaminck J, Cools P, Denwood M, Albonico M, Ame SM, et al. A general
477 framework to support cost-efficient fecal egg count methods and study design choices for large-
478 scale STH deworming programs–monitoring of therapeutic drug efficacy as a case study. PLoS
479 Negl	Trop	Dis.	Public	Library	of	Science;	2023;17.
480 https://doi.org/10.1371/JOURNAL.PNTD.0011071
481 18. Trinos JPCD, Ng-Nguyen D, Coffeng LE, Dyer CEF, Clarke N, Traub R, et al. Cost and cost-
482 effectiveness analysis of mass drug administration compared to school-based targeted preventive
483 chemotherapy for hookworm control in Dak Lak province, Vietnam. Lancet Reg Health West Pac.
484 Elsevier Ltd; 2023;41:100913. https://doi.org/10.1016/j.lanwpc.2023.100913
485 19. Cha S, Elhag MS, Lee YH, Cho DS, Ismail HAHA, Hong ST. Epidemiological findings and
486 policy implications from the nationwide schistosomiasis and intestinal helminthiasis survey in
487 Sudan. Parasit Vectors. BioMed Central Ltd.; 2019;12. https://doi.org/10.1186/s13071-019-3689-
488 z
489 20. De Neve J-W, Andriantavison RL, Croke K, Krisam J, Rajoela VH, Rakotoarivony RA, et al.
490 Health,	financial,	and	education	gains	of	investing	in	preventive	chemotherapy	for
491 schistosomiasis, soil-transmitted helminthiases, and lymphatic filariasis in	Madagascar: A
492 modeling	study.	PLoS	Negl	Trop	Dis.	United	States;	2018;12:e0007002.
493 https://doi.org/10.1371/journal.pntd.0007002
494 21. Okello WO, Okello AL, Inthavong P, Tiemann T, Phengsivalouk A, Devleesschauwer B, et al.
495 Improved methods to capture the total societal benefits of zoonotic disease control:
496 Demonstrating the cost-effectiveness of an integrated control programme for Taenia solium, soil
497 transmitted helminths and classical swine fever in northern Lao PDR. PLoS Negl Trop Dis. United
498 States; 2018;12:e0006782. https://doi.org/10.1371/journal.pntd.0006782
499 22. Thakur M, Mohammed R, Mubayi A, Edu M. Optimizing Age-Structured and Risk-Based Mass
500 Drug Administration Against Soil-Transmitted Helminthiasis in Ghana Using Cost-Effectiveness
501 Analysis. 2018; https://doi.org/10.20944/preprints201811.0111.v1
502 23. Lo NC, Lai YS, Karagiannis-Voules DA, Bogoch II, Coulibaly JT, Bendavid E, et al.
503 Assessment of global guidelines for preventive chemotherapy against schistosomiasis and soil-
504 transmitted helminthiasis: a cost-effectiveness modelling study. Lancet Infect Dis. Lancet
505 Publishing Group; 2016;16:1065–75. https://doi.org/10.1016/S1473-3099(16)30073-1
506 24. Bartsch SM, Hotez PJ, Hertenstein DL, Diemert DJ, Zapf KM, Bottazzi ME, et al. Modeling
507 the economic and epidemiologic impact of hookworm vaccine and mass drug administration
508 (MDA) in Brazil, a high transmission setting. Vaccine. Netherlands; 2016;34:2197–206.
509 https://doi.org/10.1016/j.vaccine.2016.03.018
510 25. Turner HC, Truscott JE, Fleming FM, Hollingsworth TD, Brooker SJ, Anderson RM. Cost-
511 effectiveness of scaling up mass drug administration for the control of soil-transmitted helminths:
512 a comparison of cost function and constant costs analyses. Lancet Infect Dis. United States;
513 2016;16:838–46. https://doi.org/10.1016/S1473-3099(15)00268-6
514 26. Turner HC, Truscott JE, Anderson RM. Cost-effectiveness of community-wide treatment for
515 helminthiasis. Lancet Glob Health. Elsevier Ltd; 2016;4:e156. https://doi.org/10.1016/S2214-
516 109X(15)00280-6
517 27. Lo NC, Bogoch II, Blackburn BG, Raso G, N’Goran EK, Coulibaly JT, et al. Comparison of
518 community-wide, integrated mass drug administration strategies for schistosomiasis and soil-
519 transmitted helminthiasis: A cost-effectiveness modelling study. Lancet Glob Health. Elsevier Ltd;
520 2015;3:e629–38. https://doi.org/10.1016/S2214-109X(15)00047-9
521 28. Evans D, McFarland D, Adamani W, Eigege A, Miri E, Schulz J, et al. Cost-effectiveness of
522 triple drug administration (TDA) with praziquantel, ivermectin and albendazole for the prevention
523 of neglected tropical diseases in Nigeria. Ann Trop Med Parasitol. England; 2011;105:537–47.
524 https://doi.org/10.1179/2047773211Y.0000000010
525 29. Leslie J, Garba A, Oliva EB, Barkire A, Tinni AA, Djibo A, et al. Schistosomiasis and soil-
526 transmitted helminth control in Niger: cost effectiveness of school based and community
527 distributed mass drug administration	[corrected]. PLoS Negl Trop Dis. United States;
528 2011;5:e1326. https://doi.org/10.1371/journal.pntd.0001326
529 30. Hall A, Horton S, de Silva N. The costs and cost-effectiveness of mass treatment for intestinal
530 nematode worm infections using different treatment thresholds. PLoS Negl Trop Dis. United
531 States; 2009;3:e402. https://doi.org/10.1371/journal.pntd.0000402
532 31. Brooker S, Kabatereine NB, Fleming F, Devlin N. Cost and cost-effectiveness of nationwide
533 school-based helminth control in Uganda: intra-country variation and effects of scaling-up. Health
534 Policy Plan. England; 2008;23:24–35. https://doi.org/10.1093/heapol/czm041
535 32. Guyatt H. The cost of delivering and sustaining a control programme for schistosomiasis and
536 soil-transmitted	helminthiasis.	Acta	Trop.	Netherlands;	2003;86:267–74.
537 https://doi.org/10.1016/s0001-706x(03)00047-0
538 33. Holland CV, O’Shea E, Asaolu SO, Turley O, Crompton DW. A cost-effectiveness analysis of
539 anthelminthic intervention for community control of soil-transmitted helminth infection: levamisole
540 and Ascaris lumbricoides. J Parasitol. United States; 1996;82:527–30.
541 34. Guyatt HL, Bundy DAP, Evans D. A population dynamic approach to the cost-effectiveness
542 analysis of mass anthelmintic treatment: Effects of treatment frequency on ascaris infection. Trans
543 R Soc Trop Med Hyg. 1993;87:570–5. https://doi.org/10.1016/0035-9203(93)90094-7
544 35. Mulligan JG. Economies of scale for the GI/G/c queuing model. Math Comput Model.
545 Pergamon; 1988;10:523–7. https://doi.org/10.1016/0895-7177(88)90081-7
546 36. Bartsch SM, Hotez PJ, Hertenstein DL, Diemert DJ, Zapf KM, Bottazzi ME, et al. Modeling
547 the economic and epidemiologic impact of hookworm vaccine and mass drug administration
548 (MDA) in Brazil, a high transmission setting. Vaccine. Netherlands; 2016;34:2197–206.
549 https://doi.org/10.1016/j.vaccine.2016.03.018

550

551

552	**Appendix**

553	**Appendix I: Search queries**

(Economic Evaluation*[tiab] OR Cost Effectiveness Analysis[tiab] OR Cost Effectiveness[tiab] OR Cost Effectiveness Ratio*[tiab] OR CEA[tiab] OR Cost Per Disability-Adjusted Life Years[tiab] OR DALYs[tiab] OR Cost-Per-DALY[tiab] OR Incremental Cost-Effectiveness Ratios[tiab] OR ICERs[tiab] OR Incremental Cost-Effectiveness Ratio[tiab] OR Quality- Adjusted Life Years[tiab] OR QALYs[tiab] OR Incremental Cost-Effectiveness Ratios[tiab] OR ICERs[tiab] OR Cost) AND ((((((((((((((((((((((((("intestinal worms"[Title/Abstract]) OR ("worm"[Title/Abstract])) OR ("worm infection"[Title/Abstract])) OR ("worm infection model"[Title/Abstract])) OR ("soil transmitted helminth"[Title/Abstract])) OR ("soil transmitted helminth ascaris"[Title/Abstract])) OR ("soil transmitted helminthiasis"[Title/Abstract])) OR ("ascaris"[Title/Abstract])) OR ("ascaris lumbricoides"[Title/Abstract])) OR ("roundworms"[Title/Abstract])) OR ("hookworms"[Title/Abstract])) OR ("ancylostoma duodenale"[Title/Abstract])) OR ("necator americanus"[Title/Abstract])) OR ("trichuris trichiura"[Title/Abstract])) OR ("trichuriasis"[Title/Abstract])) OR ("whipworms"[Title/Abstract])) OR ("Strongyloides stercoralis"[Title/Abstract])) OR ("s stercoralis"[Title/Abstract])) OR ("strongyloidiasis"[Title/Abstract])) OR ("soil transmitted helminth infections"[Title/Abstract])) OR ("human parasitic disease"[Title/Abstract])) OR ("intestinal nematode infections"[Title/Abstract])) OR ("sth"[Title/Abstract])) OR ("a lumbricoides"[Title/Abstract])) OR ("t trichiura"[Title/Abstract])).

**Search queries used in PubMed database**

554

555

PRISMA-ScR Checklist

[Click here to access/download](https://www2.cloud.editorialmanager.com/pntd/download.aspx?id=1494666&guid=6c521d9d-0fa2-4238-aa5f-f918abad6113&scheme=1)

[**Supporting Information** PRISMA-ScR Checklist v2.0.docx](https://www2.cloud.editorialmanager.com/pntd/download.aspx?id=1494666&guid=6c521d9d-0fa2-4238-aa5f-f918abad6113&scheme=1)
