# Supplementary material for: Cost-effectiveness of soil-transmitted helminthiasis intervention programmes: A scoping review
Source: PLoS Negl Trop Dis. 2026 May 13;20(5):e0014317. doi: 10.1371/journal.pntd.0014317 (PMC13183283; doi:10.1371/journal.pntd.0014317)
Supplement: Attachment — Submitted filename: Response to reviewers.docx [file pntd.0014317.s005.docx]

**Journal Requirements:**

1) Please upload all main figures as separate Figure files in .tif or .eps format. For more information about how to convert and format your figure files please see our guidelines: 
https://journals.plos.org/plosntds/s/figures

Author response:

In accordance with the journal’s technical requirements, we have converted all three main figures (Figure 1, Figure 2, and Figure 3) to .tif format. We have also ensured that these files are named according to the journal’s naming conventions (e.g., Fig1.tif, Fig2.tif, and Fig3.tif)

2) We have noticed that you have uploaded Supporting Information files, but you have not included a list of legends. Please add a full list of legends for your Supporting Information files after the references list.

Author response:

We thank the editor for this technical observation. In accordance with the journal’s guidelines, we have now included a ‘Supporting Information’ section immediately following the references list. This section provides a descriptive legend for each supporting information file uploaded with the submission, which is the PRISMA-ScR checklist.

3) We notice that your supplementary information is included in the manuscript file. Please remove them and upload them with the file type 'Supporting Information'. Please ensure that each Supporting Information file has a legend listed in the manuscript after the references list.

Author response:

We have removed the supporting information from the manuscript, and we will upload them separately as ‘Supporting Information’. We have also added a legend for the supporting information in the manuscript after the references list.

4) Some material included in your submission may be copyrighted. According to PLOSu2019s copyright policy, authors who use figures or other material (e.g., graphics, clipart, maps) from another author or copyright holder must demonstrate or obtain permission to publish this material under the Creative Commons Attribution 4.0 International (CC BY 4.0) License used by PLOS journals. Please closely review the details of PLOSu2019s copyright requirements here: PLOS Licenses and Copyright. If you need to request permissions from a copyright holder, you may use PLOS's Copyright Content Permission form.
Please respond directly to this email and provide any known details concerning your material's license terms and permissions required for reuse, even if you have not yet obtained copyright permissions or are unsure of your material's copyright compatibility. Once you have responded and addressed all other outstanding technical requirements, you may resubmit your manuscript within Editorial Manager. 
Potential Copyright Issues:

- Figure 2. Please (a) provide a direct link to the base layer of the map (i.e., the country or region border shape) and ensure this is also included in the figure legend; and (b) provide a link to the terms of use / license information for the base layer image or shapefile. We cannot publish proprietary or copyrighted maps (e.g. Google Maps, Mapquest) and the terms of use for your map base layer must be compatible with our CC BY 4.0 license.
Note: if you created the map in a software program like R or ArcGIS, please locate and indicate the source of the basemap shapefile onto which data has been plotted.
If your map was obtained from a copyrighted source please amend the figure so that the base map used is from an openly available source. Alternatively, please provide explicit written permission from the copyright holder granting you the right to publish the material under our CC BY 4.0 license.
If you are unsure whether you can use a map or not, please do reach out and we will be able to help you. The following websites are good examples of where you can source open access or public domain maps:
* U.S. Geological Survey (USGS) - All maps are in the public domain. (http://www.usgs.gov)
* PlaniGlobe - All maps are published under a Creative Commons license so please cite u201cPlaniGlobe, http://www.planiglobe.com, CC BY 2.0u201d in the image credit after the caption. (http://www.planiglobe.com/?lang=enl)
* Natural Earth - All maps are public domain. (<http://www.naturalearthdata.com/about/terms-of-use/>).

Author response:

Thank you for your guidance regarding the copyright requirements for the figures in the manuscript. In response to your concerns regarding Figure 2, we have replaced the original map with a new version created using open-access data to ensure full compatibility with the CC BY 4.0 licence.

**Reviewers' Comments:**

Reviewer's Responses to Questions

**Key Review Criteria Required for Acceptance?**


As you describe the new analyses required for acceptance, please consider the following:


**Methods**


-Are the objectives of the study clearly articulated with a clear testable hypothesis stated?

-Is the study design appropriate to address the stated objectives?

-Is the population clearly described and appropriate for the hypothesis being tested?

-Is the sample size sufficient to ensure adequate power to address the hypothesis being tested?

-Were correct statistical analysis used to support conclusions?

-Are there concerns about ethical or regulatory requirements being met?

Reviewer #1: Overall, this is a well written and clear paper. The results are compelling, despite the acknowledged limitations. My main concern or question regarding the methodology is whether the studies that were included based their cost-effectiveness analyses on actual (i.e., measured) costs/benefits or on assumed or estimated ones? Is there a way for the authors to ascertain this for each study and to add it to the paper? For example, the single Brazil study seems to have included inputs from several other studies (even some done in Africa).

Author response:

We thank the reviewer for this comment regarding the nature of the data inputs. We agree that distinguishing between primary (measured) and secondary (estimated) data is vital for interpreting economic evaluations. We have added a statement in the Outcomes and prioritization section to explicitly address this. Our scoping review included both program delivery cost analyses, which frequently utilize primary field-measured costs, and dynamic transmission models, which often synthesize secondary epidemiological and cost parameters from multiple sources. We believe this addition clarifies the ‘hybrid’ nature of the evidence base without requiring complete re-extraction of every individual data point. See lines 193 – 194.


Minor:
1. Figure 1 describes records marked as ineligible by "automation tools" but these tools are not described in the Methods section. Please clarify.

Author response:

We thank the reviewer for identifying this inconsistency. The mention of ‘automation tools’ in Figure 1 was a standard PRISMA 2020 template placeholder that was not sufficiently detailed in the text. We have updated the Methods section (2.3) to clarify that Mendeley Reference Manager was the primary tool used for automated duplicate removal, see lines 155 - 156. No other automated screening tools were utilized.

Reviewer #2: the objective is clear with correct statistical analysis

Author response:

Complement: No Response

Reviewer #3: The research question must be well-defined and aligned with the objectives. There is no consistency between the conclusion and the questions posed at the beginning.
Although they chose a scoping review approach, when discussing the economic models used to evaluate these studies, these models are not ultimately considered or mentioned.
The title does not accurately reflect the paper's actual purpose, and, most importantly, it fails to specify the geographical areas studied. I would suggest narrowing the scope to Sub-Saharan Africa.

Author response:

We thank the reviewer for these critical reflections. We have carefully considered the suggestion to narrow the scope to Sub-Saharan Africa, but we believe that maintaining a global scope is essential for the following reasons:

i. The primary goal of this scoping review, as per Arksey & O’Malley’s framework, was to map the global landscape and identify evidence gaps. By including all regions, we were able to provide evidence of the significant geographical bias toward Sub-Saharan Africa (59% of studies), which is itself a critical finding for global policy.
ii. Regarding the mention of economic models, we have categorized the 22 included studies by their specific modelling approach (e.g., dynamic transmission vs static cost analysis) in Section 3.3.2 and Table 2.

**Results**


-Does the analysis presented match the analysis plan?

-Are the results clearly and completely presented?

-Are the figures (Tables, Images) of sufficient quality for clarity?

Reviewer #1: 1. Line 262: please clarify that the "hookworm vaccine" is a hypothetical one, since there currently is no approved vaccine for this STH.
2. Table 2 lists the year of publication for each study, but that doesn't necessarily reflect the time period when the data were collected, since publication could have occurred years later. Would it be possible to include the dates that the studies were conducted or during which the data were collected?

Author response:

1. We thank the reviewer for this important clarification. We agree that it is critical to distinguish between existing interventions and hypothetical ones. We have revised the text to explicitly state that the hookworm vaccine evaluated in the referenced study (Bartsch et al., 2016) is a hypothetical intervention used for economic modelling and is not yet an approved or commercially available vaccine. See line 346.
2. On the point of the period when the data in the included studies were collected, we would like to clarify that for the purposes of this scoping review, the year of publication was chosen as the primary marker for the time period. The objective of a scoping review, as defined by the Arksey and O’Malley framework, is to map the existing landscape of research and identify evidence gaps rather than to perform economic trend analysis or a meta-analysis of standardized costs. Because we did not adjust for inflation or perform a comparative temporal synthesis of cost-effectiveness ratios, the specific years of data collection do not influence the findings or the categorization of the evidence presented. We believe that including the year of publication provides sufficient guidance for readers to locate the studies while maintaining the focus on the review’s core purpose.

Reviewer #2: analysis presented match the analysis plan

Author response:

Compliment. No response.

Reviewer #3: The results should be aligned with the studies. Table 1 should be improved, as it is currently redundant, and I would recommend eliminating the third column.
The map presented should have two parts: zones showing areas according to prevalence (high, medium, or low), and, either on the same or a separate map, the number of studies in choropleth maps. If the studies only considered English-language literature, I would remove the data concerning Latin America from the study to avoid drawing erroneous conclusions.

Author response:

We thank the reviewer for these specific suggestions regarding the presentation of our results. We have carefully considered each point and would like to provide the following clarifications:

1. Regarding Table 1, we have reviewed the table for redundancy. However, we believe the third column (Rationale) is essential for a scoping review, as it justifies why each study was included and how it aligns with our research objectives.
2. Regarding the maps, we understand the reviewer’s interest in seeing prevalence data; however, the primary objective of this scoping review was to map the volume of evidence and the geographical distribution of research, not to map the disease burden itself.
3. Regarding the inclusion of Latin America, we respectfully disagree with the suggestion to remove data concerning Latin America. Our research was for English-language literature, which is the international standard for global health research, but it was not limited to English-speaking countries.

**Conclusions**


-Are the conclusions supported by the data presented?

-Are the limitations of analysis clearly described?

-Do the authors discuss how these data can be helpful to advance our understanding of the topic under study?

-Is public health relevance addressed?

Reviewer #1: Overall, the conclusions appear to be supported by the data presented. Limitations are clearly stated.

Author response:

Compliment. No response

Reviewer #2: the conclusion supported by data presented, the authors discuss how data can help understanding of strategies for the control of STD.

Author response:

Compliment. No response

Reviewer #3: The study draws conclusions based on unclear information, such as defining that population-level interventions are more cost-effective than school-level interventions, when Table 1 only identifies two or three studies with those characteristics.
The limitations do not mention that the cost-effectiveness analyses that should have been used were not performed, and I believe this should be clearly stated in the limitations. As the authors themselves mention, there are significant biases in the collection and analysis of the data. I suggest improving the analysis, the formulation of the research question, and ensuring consistency throughout the article.

Author response:

We thank the reviewer for these comments; however, we would like to offer some clarifications regarding the objectives and methodology of this scoping review to address the concerns raised:

1. Regarding population-level vs school-level interventions, the conclusion that population-level interventions can be more cost-effective is not an original claim of this review. Our review found that community-based MDA was more cost-effective than school-based MDA. In a scoping review, our role is to map and summarize the results of the available literature.
2. Regarding the lack of new cost-effectiveness analyses, there appears to be a misunderstanding of our study design. As a scoping review, the established methodology (Arksey & O’Malley) prohibits the authors from performing new cost-effectiveness analyses or meta-analyses. Our purpose was to map existing analyses.
3. Regarding data biases, the ‘biases’ mentioned in our manuscript refer to the limitations inherent in the primary studies we reviewed. Highlighting these gaps is a strength of a scoping review, as it identifies where future research needs to improve. It does not indicate a flaw in our data collection process.

**Editorial and Data Presentation Modifications?**


Use this section for editorial suggestions as well as relatively minor modifications of existing data that would enhance clarity. If the only modifications needed are minor and/or editorial, you may wish to recommend “Minor Revision” or “Accept”.

Reviewer #1: 1. Lines 91-93: how does coformulation of albendazole and ivermectin in a fixed-dose combination provide evidence of effectiveness? I think that what the authors mean to say is that recent evidence from a Phase 2/3 trial (published in TLID) showed improved efficacy of the FDC vs single-dose ALB against T. trichiura. However, the sentence as currently worded does not state this and should be revised for clarity.

Author response:

We thank the reviewer for this excellent clarification. We agree that the co-formulation itself is not evidence, but rather the vehicle for delivering the improved efficacy demonstrated in recent clinical trials. We have revised the sentence to more accurately reflect that recent Phase 2/3 trial data demonstrate the superior efficacy of the albendazole and ivermectin FDC compared to single-dose albendazole, particularly against *T. trichiura.* This change ensures that we are correctly distinguishing between the formulation and the clinical outcomes. See lines 91 – 95.

2. Line 166: should this be "ratio" instead of "ration" (ICER definition)?

Author response:

We’ve changed “ration” to “ratio” in the manuscript. See line 170.

Reviewer #2: These data could have been very useful in general. Because of the limitation to English litterature its sound that in area where STD and others parasite like Loa loa co exist. strategies may be different. I will suggest that the authors expand discussion on this particular situation ( IVM= ivermetin, may be use with caution in loiasis endemic countries ....) where economic benefits of combined ALB/IVM needs further studies.

Author response:

We agree that the economic and safety trade-offs in these specific geographical contexts are critical. However, we would like to clarify that the objective of this scoping review was not to evaluate the clinical safety or specific cost-effectiveness of an albendazole/ivermectin (ALB/IVM) regimen itself. Rather, our goal was to map the broad landscape of strategies currently represented in the literature and identify evidence gaps. As noted in our results, very few studies have specifically modeled the economic implications of integrated or FDC-based programs.

Reviewer #3: (No Response)

**Summary and General Comments**


Use this section to provide overall comments, discuss strengths/weaknesses of the study, novelty, significance, general execution and scholarship. You may also include additional comments for the author, including concerns about dual publication, research ethics, or publication ethics. If requesting major revision, please articulate the new experiments that are needed.

Reviewer #1: (No Response)

Reviewer #2: This study map the area covered by mass drug administration for STD control in the world. Its show economic value of community-based control over school children based control. its demonstrate that community-based control is more economical than school children-based strategy. We suggest to the authors to expand their discussion in area where STD co-exist whit filaria Loa loa

Author response:

We indicated that community-based interventions are more cost-effective than school-based strategies. Our focus is not on the diseases specifically, but rather on the intervention, whether school-based, community-based, or integrated, and their cost-effectiveness.

Reviewer #3: I believe that while the article addresses an important topic such as the cost-effectiveness of programs, it fails to integrate the necessary methods for conducting such analyses through a systematic review. I suggest that the title be revised, clearly stating that it is merely a description and not an analysis, as is the conclusion reached.

Author response:

We thank the reviewer for this comment. We would like to politely clarify that this study was designed and conducted specifically as a Scoping Review, following the established Arksey and O’Malley framework and the PRISMA-ScR (Scoping Review) guidelines. A scoping review is fundamentally different from a Systematic Review in its intent: while a Systematic Review seeks to answer a narrow clinical question by analysing data, a Scoping Review aims to map the volume, nature, and characteristics of a broad body of research to identify evidence gaps. Therefore, the descriptive nature of our results is the standard and appropriate outcome for this methodology.

Regarding the title, we believe that ‘Scoping Review’ is the most accurate and transparent term, as it immediately informs the reader that the paper provides a high-level map of the field’s evidence rather than secondary statistical analysis of the costs themselves.

PLOS authors have the option to publish the peer review history of their article ([what does this mean?](https://journals.plos.org/plosntds/s/editorial-and-peer-review-process#loc-peer-review-history)). If published, this will include your full peer review and any attached files.


If you choose “no”, your identity will remain anonymous but your review may still be made public.


**Do you want your identity to be public for this peer review?** For information about this choice, including consent withdrawal, please see our [Privacy Policy](https://www.plos.org/privacy-policy).

Reviewer #1: No

Reviewer #2: No

Reviewer #3: No

[NOTE: If reviewer comments were submitted as an attachment file, they will be attached to this email and accessible via the submission site. Please log into your account, locate the manuscript record, and check for the action link "View Attachments". If this link does not appear, there are no attachment files.]

**Figure resubmission:**

While revising your submission, we strongly recommend that you use PLOS’s NAAS tool (https://ngplosjournals.pagemajik.ai/artanalysis) to test your figure files. NAAS can convert your figure files to the TIFF file type and meet basic requirements (such as print size, resolution), or provide you with a report on issues that do not meet our requirements and that NAAS cannot fix.

After uploading your figures to PLOS’s NAAS tool - https://ngplosjournals.pagemajik.ai/artanalysis, NAAS will process the files provided and display the results in the "Uploaded Files" section of the page as the processing is complete. If the uploaded figures meet our requirements (or NAAS is able to fix the files to meet our requirements), the figure will be marked as "fixed" above. If NAAS is unable to fix the files, a red "failed" label will appear above. When NAAS has confirmed that the figure files meet our requirements, please download the file via the download option, and include these NAAS processed figure files when submitting your revised manuscript.

**Reproducibility:**

To enhance the reproducibility of your results, we recommend that authors of applicable studies deposit laboratory protocols in protocols.io, where a protocol can be assigned its own identifier (DOI) such that it can be cited independently in the future. Additionally, PLOS ONE offers an option to publish peer-reviewed clinical study protocols. Read more information on sharing protocols at https://plos.org/protocols?utm_medium=editorial-email&utm_source=authorletters&utm_campaign=protocols
